# The role of attention in subliminal semantic processing: A mouse tracking study

**DOI:** 10.1371/journal.pone.0178740

**Published:** 2017-06-13

**Authors:** Kunchen Xiao, Takashi Yamauchi

**Affiliations:** Department of Psychology, Texas A&M University, College Station, Texas, United State of America; University of Akron, UNITED STATES

## Abstract

Recent evidence suggests that top-down attention facilitates unconscious semantic processing. To clarify the role of attention in unconscious semantic processing, we traced trajectories of the computer mouse in a semantic priming task and scrutinized the extent to which top-down attention enhances unconscious semantic processing in four different stimulus-onset asynchrony (SOA: 50, 200, 500, or 1000ms) conditions. Participants judged whether a target digit (e.g., “6”) was larger or smaller than five, preceded by a masked priming digit (e.g., “9”). The pre-prime duration changed randomly from trial to trial to disrupt participants’ top-down attention in an uncued condition (in a cued condition, a green square cue was presented to facilitate participants’ top-down attention). The results show that top-down attention modifies the time course of subliminal semantic processing, and the temporal attention window lasts more than 1000ms; attention facilitated by the cue may amplify semantic priming to some extent, yet the amplification effect of attention is relatively minor.

## Introduction

### Unconscious processing can be elaborate and flexible

Unlike the traditional view that unconscious cognition is stereotypical and independent of cognitive control [1, 2, 3, 4], recent evidence shows that subliminal processing can occur at a semantic level and be modulated by cognitive control [5, 6, 7, 8]. A typical form of cognitive control is top-down attention, which is the cognitive process of allocating limited processing resources and selectively concentrating on a particular aspect of information while ignoring other perceivable information. Though attention is often confounded with consciousness, they are not identical; attention is the mechanism allocating limited cognitive resource to selectively polarize particular processing, while consciousness is the mental status of subjective awareness [6, 7]. Recent studies suggest that top-down attention modulates subliminal processing [6, 7, 9].

This radical conceptual shift in conscious and unconscious processing stems from evidence accrued in “masked semantic priming” studies [5, 9]. In a typical study [9], participants judged whether a target digit (e.g., “1”) was larger or smaller than five, preceded by a masked priming digit (e.g., “9”). The pre-prime duration changed randomly from trial to trial to disrupt participants’ top-down attention because it was impossible to predict when a target number would appear (i.e., uncued condition). In a cued condition, however, a green square cue was presented for 200ms to signal an upcoming target number, facilitating participants’ top-down attention (the interval between the cue and target was fixed at 384ms). Results showed that subliminal congruency effects were present in the cued condition while absent in the uncued condition, indicating that top-down attention amplified subliminal semantic processing. This notion is supported by other studies such as participants correctly reporting locations of subliminal visual stimuli only when top-down attention is present, and subliminal comprehension of natural scenes requires the assistance of top-down attention [10].

Nevertheless, evidence for the role of attention in subliminal semantic processing is limited, and effect sizes of masked priming are often small and difficult to be replicated [11]. Other studies also emphasized a relatively minor role that top-down attention plays and suggest that subliminal semantic priming does occur in near absence of top-down attention [12–15].

One reason for the paucity of reliability in masked priming is the way that priming effects have been measured. Temporal measures, such as response time, only make use of two data points in each trial: the onset time and the end time; any intervening factors that occur in-between can be glossed over. Thus, the response time is not very informative about dynamic decision-making processes unfolding in real-time. The current study introduces an alternative method—tracking trajectories of the computer mouse in choice-reaching—to investigate the role of top-down attention in unconscious semantic processing.

### Assessing unconscious processing with a mouse tracking method

The mouse tracking method records the computer mouse movement trajectories during decision-making and provides viable information about unconscious semantic processing [16–20]. In each trial, participants use the computer mouse to make a response (reaching a target button and click the button to indicate a response). When participants are conducting this choice-reaching task, the x-y coordinate of the cursor location on the screen is recorded every 15ms to track the trajectory of cursor motion [21–25]. The mouse tracking method records real-time temporal-spatial information underlying participants’ dynamic decision-making processes [26–27]. By examining real-time features of decision-making, temporal-spatial data provide insights into subliminal semantic processing [17, 28, 29].

Research has shown that hand motion in choice-reaching tasks shows participants’ subliminal decision-making processes [30, 31]. Lately, a number of studies have adopted cursor trajectory measures to investigate elaborate unconscious processing. For instance, perceptual and semantic processing can be separated by different cursor motion patterns [32–34]. Congruency effects can be assessed by attractions toward unintended choices of cursor movements [35–39].

Moreover, a number of studies have shown the advantage of the mouse tracking measure over the response time measure [18–20]. This advantage is pronounced particularly in unconscious processing [19, 40, 41]. Research comparing the cursor trajectory measure and response latency measure also indicates that the effect size for priming obtained from the mouse tracking measure is significantly larger than that by response time measures [18, 20].

**Fig 1:**
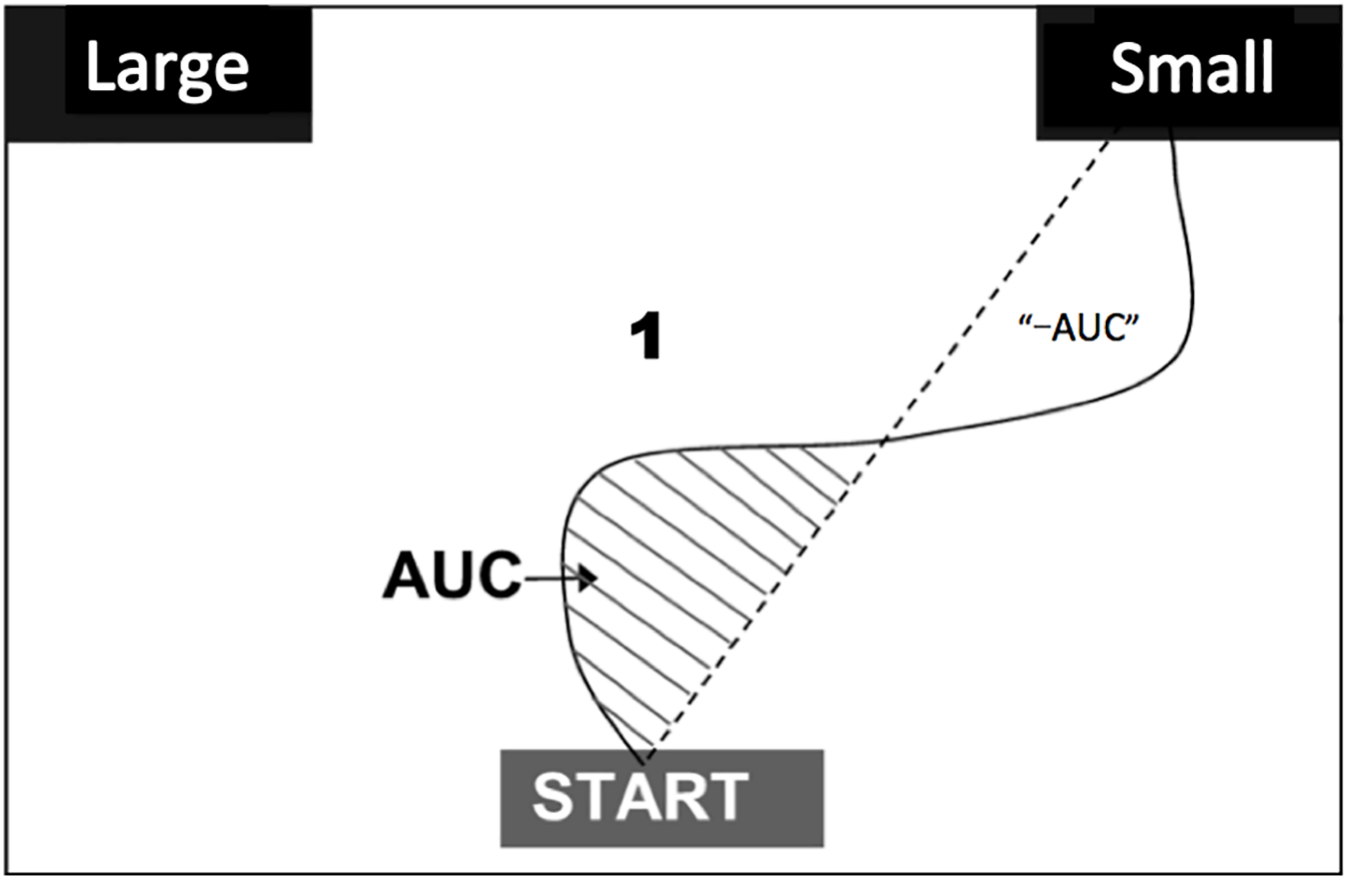
Illustration of the area under the curve (AUC). In this example, participants judge whether the number “1” presented at the center of the screen is larger or smaller than five. The curve in the figure represents a trajectory of the cursor from the onset position (“START” button) to the ending position (“Small” button). The dashed straight line represents the “ideal path”. The AUC (measured by the number of pixels) is the shaded area circumscribed by the ideal path and the actual trajectory that exceeds the ideal path toward the *unselected* option (shaded area). Any area exceeds the idea path toward the *selected* option will be subtracted as “minus AUC”.

In our cursor movement analysis, the area under the curve (AUC) of a trajectory of the computer cursor is calculated in each trial as the area between a straight line connecting the onset location (the center of the “Start” button on the bottom of the screen) and the ending location (one of the two buttons at the top left or right corner of the screen; where participants click the button is defined as the ending point) and the actual trajectory that goes beyond the straight line toward the unselected choice; any area exceeds the idea path toward the selected option will be subtracted from the AUC (Fig 1). Smaller AUCs indicate that the trials are more certain to respond to, while larger AUCs more uncertain [26]. To obtain a cursor motion trajectory, the x-y coordinate of the cursor location on the screen is recorded as one data point every 20ms; since participants’ response trajectories have different lengths of duration, to be able to calculate the AUC, it is necessary to normalize the timing of all trajectories; by applying a linear interpolation method, all the data points are standardized into 100 steps for each trial. The AUC is measured by the number of pixels. Although the interpretation of AUC data is sometimes similar to that of response time, they are two different measurements; given the normalization process in calculating AUC, the AUC focuses on overall deviation during choice-reaching rather than timing information [26].

### Overview of the current experiment

Employing the mouse tracking method, we investigate the influence of top-down attention on subliminal semantic processing in the number judgment task applied by Naccache, Blandin, and Dehaene (2002) and Xiao and Yamauchi (2015) [9, 19]. In these studies, participants were asked to judge whether a target digit was larger or smaller than five, and top-down attention was manipulated by contrasting cued and uncued conditions. In the cued condition, a green square cue was presented to facilitate attention. While in the uncued condition, attention was disrupted by random pre-target durations. Both Naccache et al. (2002) and Xiao and Yamauchi (2015) found larger priming effects in the cued condition than in the uncued condition and suggested that top-down attention amplified priming; specifically, when participants saw the cue and prepared themselves for the upcoming target, they opened a temporal attention window, which lasted from the onset of the cue until making a response to the target; thus, the subliminal semantic processing of the masked prime presented between the cue and the target was enhanced by the temporal attention window [9, 19]. However, these studies applied fixed 100ms stimulus-onset asynchrony (SOA), which only provides a partial time course of subliminal semantic processing. This constraint made it unclear how long the temporal attention window would last and how exactly attention influences subliminal semantic processing.

The current study extends the Xiao and Yamauchi (2015) experiment by systematically manipulating the SOA among four between-subjects conditions (50ms, 200ms, 500ms, 1000ms) to probe the time course of the temporal attention window. In doing so, we address two unanswered questions: (1) how long is the lifespan of the temporal attention window; (2) how exactly attention influences subliminal semantic processing.

The temporal attention window (TAW) hypothesis [5, 9, 42] suggests that the top-down attention window lasts for approximately 500ms (i.e., the duration from the onset of the cue to the onset of the prime), which amplifies subliminal semantic processing. Yet, it is unclear whether the temporal attention window covers the prime only or covers both the prime and the target, because interval between the prime and target was very brief (i.e., 100ms SOA) in the Naccache et al. (2002) [9] and Xiao & Yamauchi (2015) studies [19]. In the current experiment, the duration from the onset of the cue to the onset of the target (i.e., the lifespan of the temporal attention window) is more than one second in the 500ms and 1000ms SOA conditions. If the temporal attention window lasts only a few hundred milliseconds, priming should be weakened substantially for 500ms and 1000ms SOA. If the temporal attention window lasts for more than 1000ms, semantic priming should be observed for 500ms SOA (or even for 1000ms SOA).

**Fig 2:**
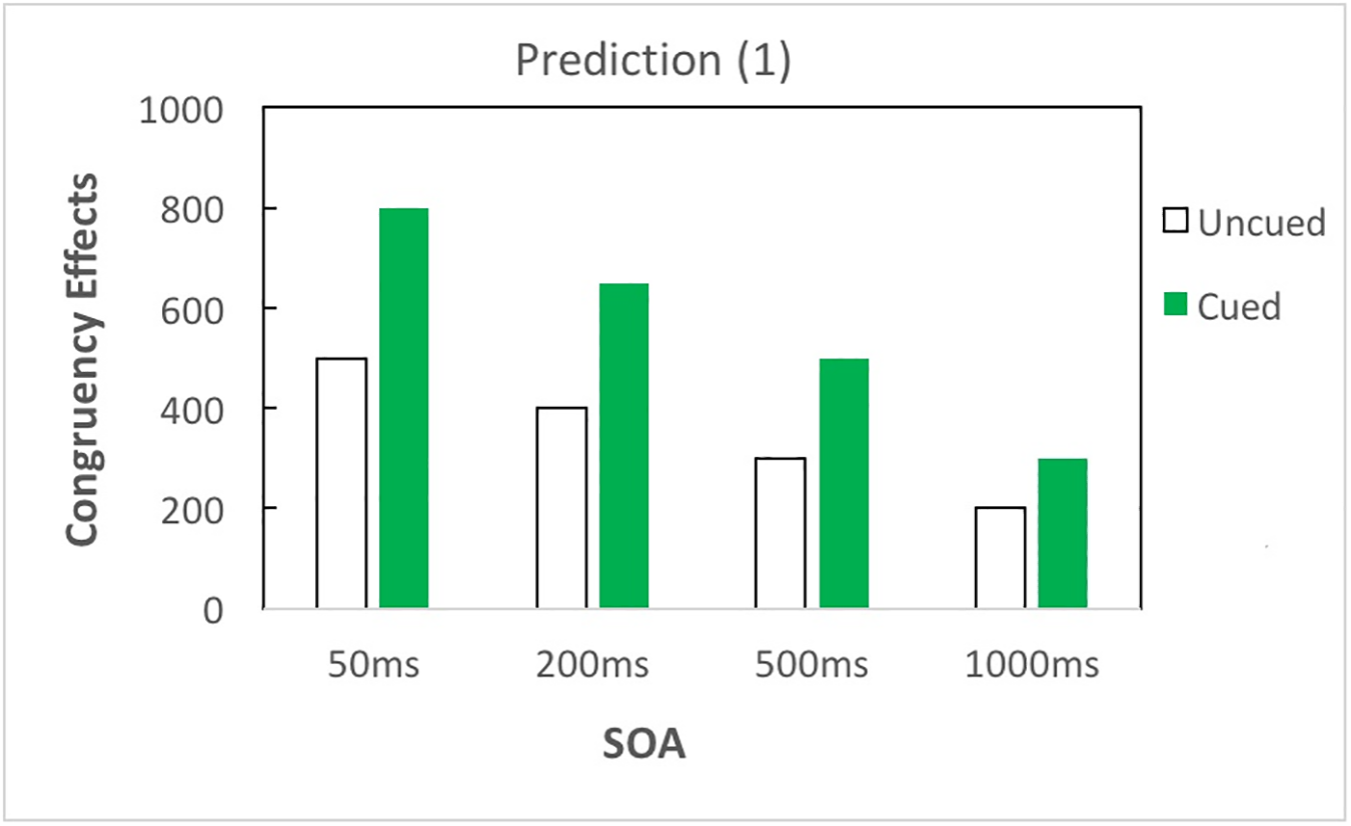
Prediction (1). Top-down attention amplifies priming without changing the time course of semantic processing. If top-down attention amplifies priming without changing the time course of subliminal semantic processing, cued trials would elicit larger congruency effects (i.e., AUC _incongruent_—AUC _congruent_) as compared to uncued trials overall; yet both cued and uncued trials would follow analogous time course—priming would occur earlier and decrease linearly as SOA increases. The AUC is measured by the number of pixels.

**Fig 3:**
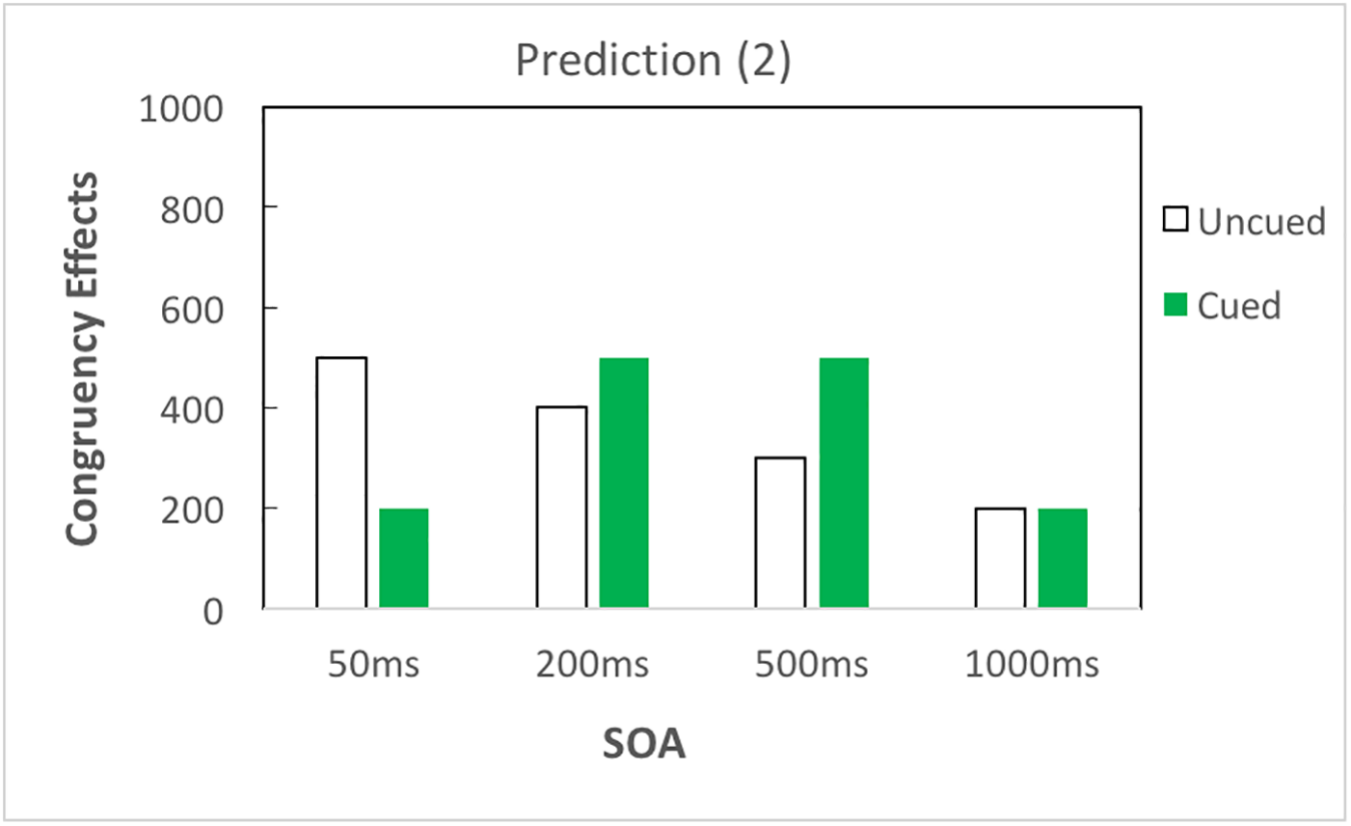
Prediction (2). Top-down attention modifies the time course of subliminal semantic processing without amplifying priming. If top-down attention only modifies the time course of subliminal semantic processing without amplifying priming, cued and uncued trials diverge only in their time course of priming but overall congruency effects remain equal.

**Fig 4:**
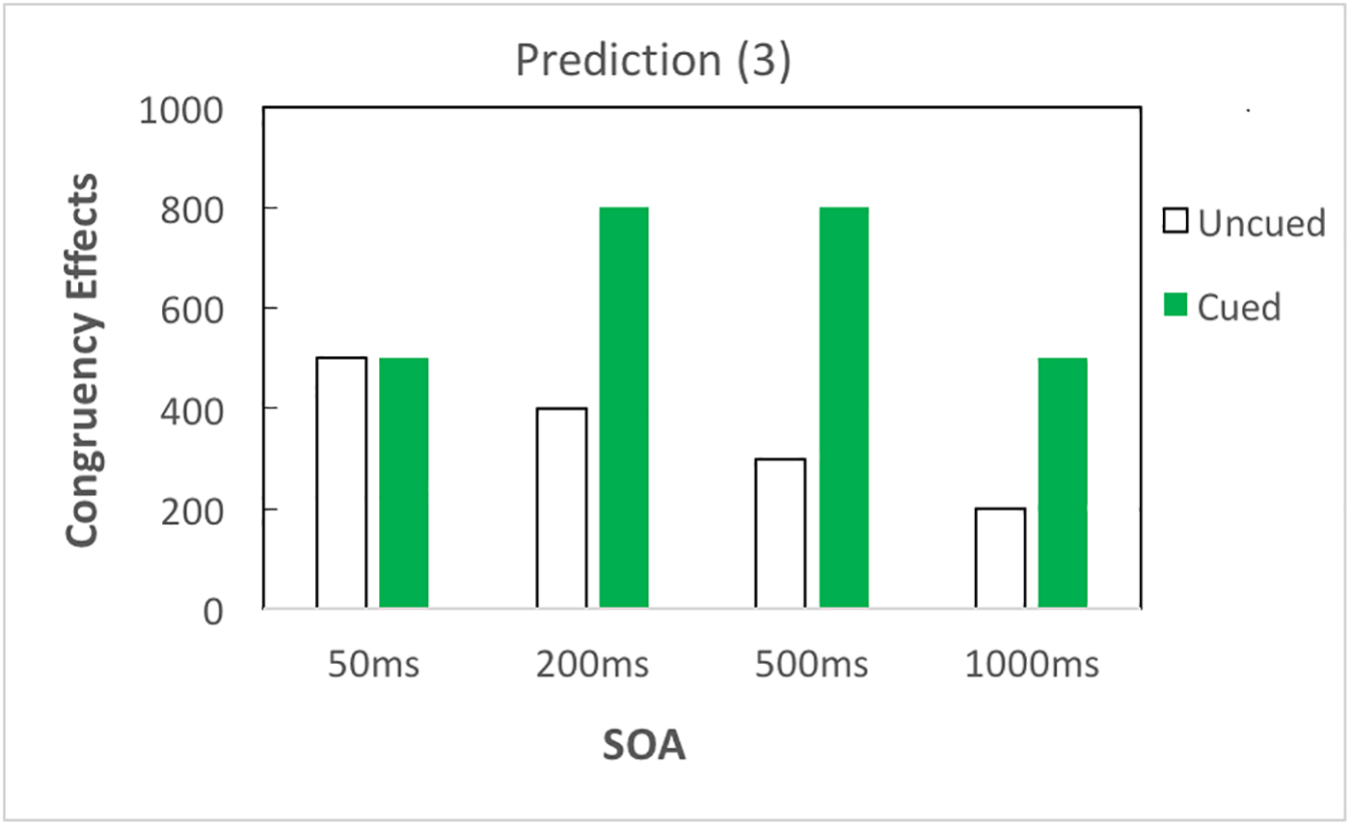
Prediction (3). Top-down attention amplifies priming and modifies the time course of subliminal semantic processing. If top-down attention amplifies priming and modifies the time course of subliminal semantic processing, cued trials would elicit larger congruency effects as compared to uncued trials overall; yet cued and uncued trials would follow different time courses. For example, in uncued trials, priming occurs earlier and decreases linearly as SOA increases; while in cued trials, priming arises and decreases in a quadratic manner (i.e., an inverted U curve).

With respect to the impact of top-down attention, if attention amplifies semantic priming without changing the time course, there would be larger congruency effects in cued than in uncued trials, and both cued and uncued trials would follow analogous time courses; that is, priming would occur earlier and decrease linearly as SOA increases (Fig 2). If top-down attention merely modifies the time course of priming without amplifying priming, the amplification effect of attention reported by Naccache et al. (2002) [9] could be explained by delayed time course of priming influenced by attention; in this case, congruency effects in the cued condition would emerge and fade away later, as compared to the uncued condition (Fig 3). Besides, it is also possible that top-down attention both amplifies priming and modifies the time course of priming (Fig 4).

In brief, top-down attention could influence subliminal semantic processing in three possible manners: (1) top-down attention amplifies semantic priming without changing the time course of priming (Fig 2); (2) top-down attention modifies the time course of priming without amplifying priming (Fig 3); and (3) top-down attention amplifies and modifies the time course of priming (Fig 4). To test these predictions, our results are reported in three subsections: overall results of three-way ANOVA, contrast analyses to investigate the time course of semantic priming, and additional analyses to clarify the role of attention in subliminal semantic processing.

## Methods

**Fig 5:**
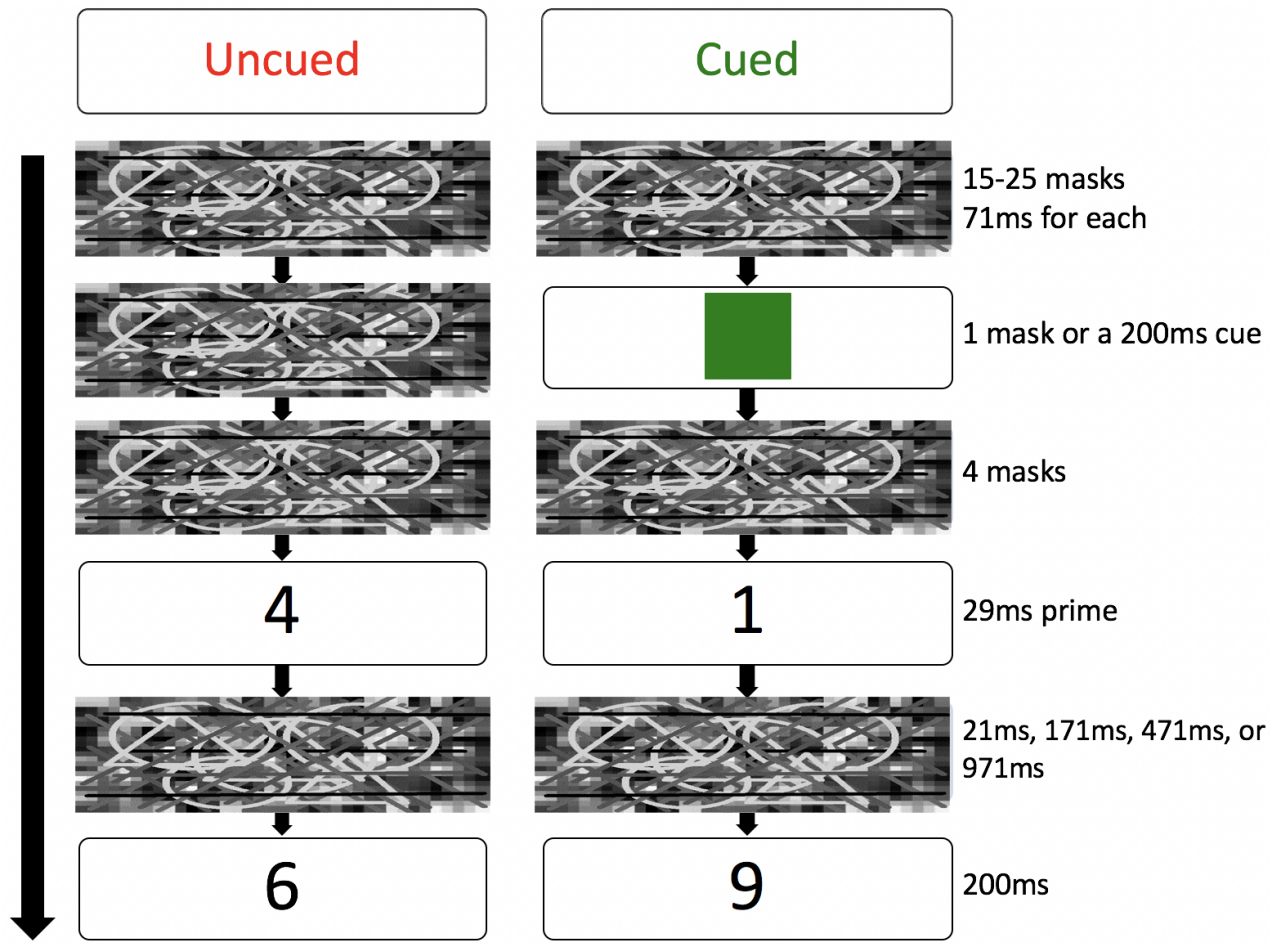
Experimental procedure. The only difference between the four SOA conditions is the post-prime mask duration: 21ms, 171ms, 471ms, or 971ms.

The current experiment consisted of two phases: a numeric judgment task followed by an awareness test. In the numeric judgment task, participants were required to decide whether a target digit (e.g., 9) was larger or smaller than 5. A priming digit (e.g., 1) was flashed subliminally for 29ms followed by a mask right before the presentation of the target digit. As in the Naccache et al. (2002) study, there were two within-subjects conditions—an uncued condition and a cued condition. In the uncued condition, the pre-target duration changed randomly from trial to trial, making it difficult for participants to anticipate when the target would appear. While in the cued condition, a green square cue was presented for 200ms to signal the upcoming target, which facilitated participants’ top-down attention to stimuli (Fig 5).

In the awareness test, participants received the same set of trials as they received in the numeric judgment task. However, participants were instructed to decide whether the priming digit, instead of the target digit, was larger or smaller than five by clicking either the “Large” or “Small” button just as they did in the numeric judgment task. Except for this single point, the awareness test was identical to the numeric judgment task.

There were 288 trials in the numeric judgment task (96 cued trials and 192 uncued trials), prior to which 96 trials (32 cued trials and 64 uncued trials) were given for practice. The reason for having less cued trials is to make the cued trials stand out, thus the “unusual” cued trials facilitates participants’ attention. In the awareness test, there were 192 trials (64 cued trials and 128 uncued trials).

### Participants

A total of 183 undergraduate students from Texas A&M University participated in this experiment for course credits. One participant in the 50ms condition, one participant in the 200ms condition, two participants in the 500ms condition, and one participant in the 1000ms condition were excluded from data analysis because their response accuracy was lower than 80%. In total, the data from 178 participants was analyzed (*n* = 45 in the 50ms SOA condition; *n* = 45 in the 200ms SOA condition; *n* = 45 in the 500ms SOA condition; and *n* = 43 in the 1000ms SOA condition).

This study was approved by Texas A&M University (IRB#2012-0451M). A written informed consent was obtained from all participants before starting the experiment.

### Materials and apparatus

Primes and targets were Arabic digits (i.e., 1, 4, 6, or 9). Stimuli were presented on a 70 Hz monitor with 1024 × 768 resolution, and the experimental procedure was controlled by a customized computer program developed with C#.

### Procedure

Participants were randomly assigned to one of four SOA conditions: 50ms, 200ms, 500ms, or 1000ms. The procedure was identical to the second experiment in the Naccache et al. (2002) study except for different SOAs (Fig 5). For each trial, a mask (71ms duration) was presented one after another seamlessly in random frequencies ranging from 15 to 25, followed by either a green square cue lasting for 200ms (cued trials) or the same mask for 71ms (uncued trials). Then four masks were presented, followed by a priming digit (i.e., 1, 4, 6, or 9) for 29ms. Following that, a post-mask was presented for 21ms (50ms SOA), 171ms (200ms SOA), 471ms (500ms SOA), or 971ms (1000ms SOA). Then a target digit (i.e., 1, 4, 6, or 9) was shown for 200ms.

Participants’ task was to decide whether the target digit was smaller or larger than five. Participants responded by clicking either the “Small” or “Large” button on the top left or right corner of the screen using the computer mouse. Participants were required to respond as accurately and quickly as possible. There were 96 cued and 192 uncued trials in this numeric judgment task; the number of congruent and incongruent trials was equal. Trials were presented in a random order for each participant. Prior to the numeric judgment task, participants performed 96 trials for practice.

In the awareness test, the identical set of trials that were used in the numeric judgment task was given. However, instead of judging the target digit, participants were explicitly informed that there was a masked digit (“prime”) prior to the target digit in each trial, and participants were required to try their best to judge whether the priming digit was smaller or larger than 5 and select either the “Small” or “Large” button accordingly. The awareness test is conservative and rigorous because the visibility of primes is lower in the main test than that in the awareness test; when participants’ judgment of primes in the awareness is at a chance level (*d’* = 0), then it is even harder for participants to be able to see primes in the main task [5, 43]. There were 192 trials in the awareness test. To assess the visibility of primes, we calculated *d’* in the awareness test [5, 9, 43–45]. Specifically, we defined “hit” as participants selecting “Large” when the priming digit was indeed larger than 5 and “false alarm” as participants choosing “Large” when the prime was actually smaller than 5. The influence of prime visibility on priming was investigated by regression analyses with congruency effects (i.e., AUC _incongruent_—AUC _congruent_) on *d’*s.

“Subliminal semantic processing” is operationally defined as the presence of congruency effect (statistically smaller AUCs in the congruent condition than in the incongruent condition) when primes were not visible. In the awareness test, the visibility of a prime is assessed by the *d’*; zero *d’* means participants’ judgment of the *prime* was at a chance level, thus the prime is barely visible [43–45]. Significant congruency effects at null *d’* is the conventional definition of “subliminal semantic processing” in research on unconscious processing in number judgment, lexical categorization, and picture categorization [5, 11, 42, 43].

### Design

The experiment had a 4 (SOA: 50ms, 200ms, 500ms, 1000ms; between-participants) × 2 (cue: cued, uncued; within-participants) × 2 (congruency: congruent, incongruent; within-participants) factorial design. The dependent measure was AUC (area under the curve).

Because the core of this study is the congruency effect, we focus on the main effect of congruency and interaction effects involving congruency (i.e., congruency × cue; congruency × SOA; congruency × cue × SOA) to facilitate exposition.

## Results

In this section, we first report overall results of ANOVA and congruency effects, then investigate the time course of semantic priming using contrast analysis. In the last section, we reported a priori planned t-test separately in cued and uncued conditions to examine the role of attention in semantic priming.

### Overall results

As expected, a three-way ANOVA (congruency × cue × SOA) showed a significant main effect of congruency; *F*(1, 174) = 23.00, *MSE* = 24457561.60, *p* < 0.001, partial ɳ^2^ = 0.12. AUCs of congruent trials were significantly smaller than those of incongruent trials (congruent trials, *M* = 5713.03, *SD* = 4317.69; incongruent trials, *M* = 6046.36, *SD* = 4417.42).

Also, there was a significant two-way (congruency × SOA) interaction effect (*F*(3, 174) = 4.73, *MSE* = 5025525.58, *p* < 0.01, partial η^2^ = 0.08), implying that semantic priming followed specific time courses (See Table 1 for details). The two-way interaction between congruency and cue was marginally significant; *F*(1, 174) = 3.12, *MSE* = 161981516.9, *p* = 0.08, partial η^2^ = 0.02. The exact role of top-down attention facilitated by the cue will be investigated later.

**Table 1:** Congruency effects (mean AUCs) with cue and uncued conditions collapsed.

SOA	N	Congruent	Incongruent	Difference	t	P-value	Cohen’s d
50ms	45	4604.24	5051.14	446.90	2.23	0.03	0.33
200ms	45	5789.97	6433.69	643.72	4.31	<0.001	0.64
500ms	45	5417.11	5776.14	359.03	1.90	0.06	0.28
1000ms	43	7102.55	6965.29	-137.26	-0.82	0.42	0.13

The AUC (area under the curve) is measured by the number of pixels.

The three-way interaction (congruency × cue × SOA) was not significant; *F*(3, 174) = 1.25, *MSE* = 161981516.9, *p* = 0.30, partial η^2^ = 0.02. The main effect of SOA was not significant; *F*(1, 174) = 1.93, *MSE* = 74862845.0, *p* = 0.13, partial η^2^ = 0.03. The main effect of cue was not significant; *F*(1, 174) = 1.73, *MSE* = 24457561.6, *p* = 0.19, partial η^2^ = 0.01. The interaction between SOA and cue was not significant; *F* < 1.0.

### Time course of priming: Contrast analysis

**Fig 6:**
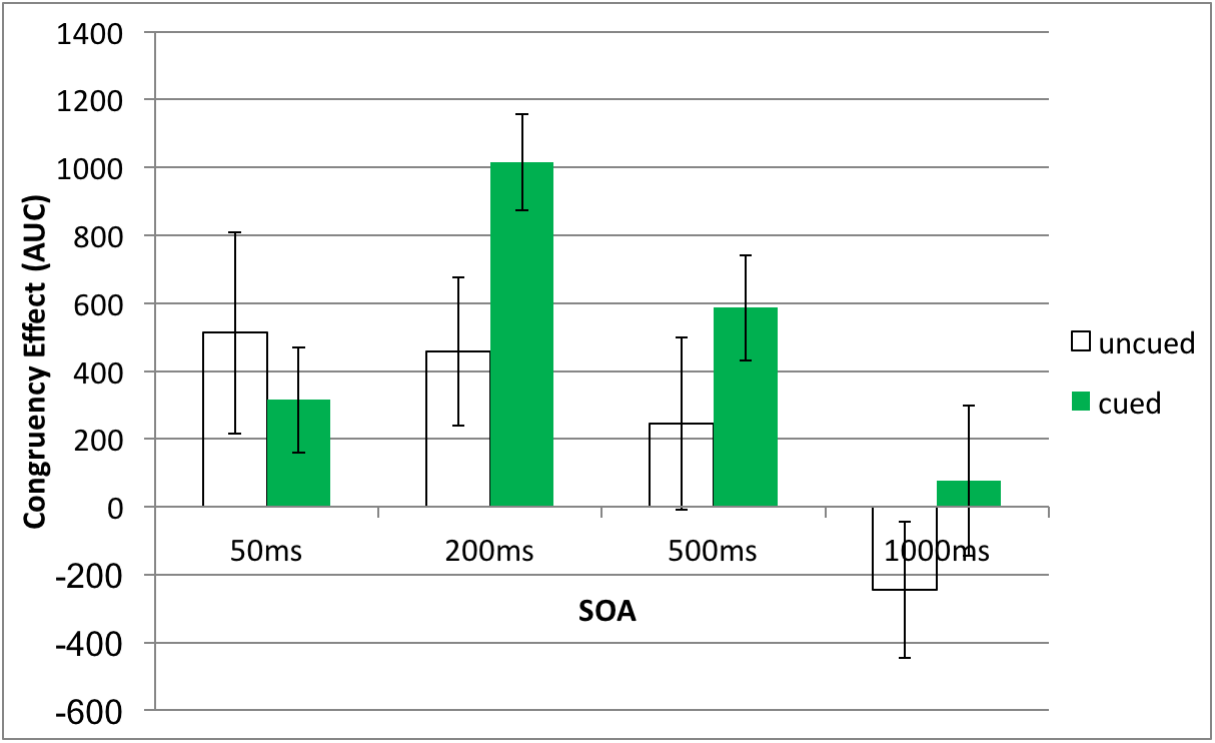
Congruency effects (AUC _incongruent_—AUC _congruent_) in the four SOA conditions in uncued and cued conditions. The X-axis denotes the four SOA conditions, while the Y-axis is the number of pixels. The error bars are two standard errors in length.

To further clarify the time course of semantic priming, we calculated the congruency effect (i.e., AUC _incongruent_—AUC _congruent_) for each SOA and performed contrast analysis separately in the cued and uncued conditions. These analyses revealed that the time course of semantic priming was modified by top-down attention (Fig 6). Specifically, in the cued condition, a quadratic trend (contrast coefficients (-1, 1, 1, -1)) was significant (*t*(174) = 3.56, *p* < 0.001) whereas a linear trend (contrast coefficients (3, 1, -1, -3) was not (*t*(174) = 1.50, *p* = 0.14). The opposite trend appeared in the uncued condition; a quadratic trend (contrast coefficients (-1, 1, 1, -1)) was not significant (*t*(174) = 0.88, *p* = 0.38) whereas a linear trend (contrast coefficients (3, 1, -1, -3) was significant (*t*(174) = 2.24, *p* = 0.03).

Thus, cued and uncued trials exhibited different time courses of semantic priming. Congruency effects occurred earlier and decreased linearly in the uncued condition. While in the cued condition, the congruency effect was initially small but emerged robust in the middle (200ms and 500ms SOA) before eventually dissipated. Thus, top-down attention modified the time course of semantic priming.

It should be noted that the normalization process in calculating AUCs eliminates the timing of raw responses because raw trajectories was cast into 100 standardized temporal bins to obtain the spatial deviations from the “ideal” straight line. Thus, the length of temporal bins was different between trials due to different response times. To recover the timing at the response level, we plot average raw trajectories of x-coordinates over raw response times in S1 File. The plots show that the x-coordinates of trajectories between congruent and incongruent trials diverge very early and then converge in several hundred milliseconds as the response goes on, reflecting participants’ real-time adjustment of choice-reaching motion.

### The role of attention in semantic priming

As reported earlier, there was marginally significant main effect of cue; *F*(1, 174) = 3.13, *MSE* = 1861856.5, *p* = 0.08, partial η^2^ = 0.02. The congruency effect is significantly larger in the cued than uncued condition when the SOA is 200ms (Table 2); the difference of congruency effects between cued and uncued conditions was not significant for any other SOA (Table 2). Additional planned t-tests applied separately for the cued and uncued conditions showed significant congruency effects primarily in 200ms, and 500ms SOAs in the cued condition (Table 3). In the uncued condition, congruency effects were generally minor (Table 4).

**Table 2:** Comparisons of congruency effects (AUC) between the cued and uncued conditions.

SOA	N	Cued	Uncued	Difference	t	P-value	Cohen’s d
50ms	45	314.75	512.98	-198.23	-0.57	0.57	0.08
200ms	45	1015.93	457.62	558.31	2.06	<0.05	0.31
500ms	45	586.77	245.16	341.61	1.29	0.20	0.19
1000ms	43	77.36	-244.57	321.93	1.21	0.23	0.18

The congruency effect is the AUC difference between congruent and incongruent trials (i.e., AUC _incongruent_—AUC _congruent_).

**Table 3:** Congruency effects (AUC) in the cued condition.

SOA	N	Congruent	Incongruent	Difference	t	P-value	Cohen’s d
50ms	45	4731.15	5045.9	314.75	2.02	<0.05	0.30
200ms	45	5460.02	6475.95	1015.93	7.18	<0.001	1.07
500ms	45	5215.27	5802.04	586.77	3.78	<0.001	0.56
1000ms	43	6859.38	6936.74	77.36	0.35	0.73	0.05

**Table 4:** Congruency effects (AUC) in the uncued condition.

SOA	N	Congruent	Incongruent	Difference	t	P-value	Cohen’s d
50ms	45	4540.79	5053.77	512.98	1.73	0.09	0.26
200ms	45	5954.95	6412.57	457.62	2.09	0.04	0.31
500ms	45	5518.03	5763.18	245.15	0.96	0.34	0.14
1000ms	43	7224.14	6979.57	-244.57	-1.21	0.23	0.18

We did not apply any corrections (e.g., Bonferroni Correction) for multiple t-tests because congruency effects in different SOAs are positively correlated; in this context, the Bonferroni Correction is over conservative and increases the probability of false negative. The impact of top-down attention on subliminal semantic processing demonstrated by Naccache et al. (2002) could be trivially “refuted” by simply increasing the number of comparisons with multiple SOA conditions if corrections were introduced. Moreover, our contrast analyses are consistent with the t-tests. Taken together, our results suggest that top-down attention modified the time course of priming; attention seemed to amplify congruency effects to some extent; yet the amplification effect of attention was not as salient as reported by previous research (Naccache et al., 2002) [9].

### Awareness analysis

To examine whether the observed semantic priming occurred at a subliminal level or not, we calculated *d’* in the awareness test to measure the visibility of the masked primes [43, 45]. Here, we defined “hit” as participants making a “large” response when the priming digit was indeed larger than 5 and “false alarm” as participants making a “large” response when the prime was, in fact, smaller than 5.

The *d’* analysis suggests that the masked primes were hardly visible; the *d’*s were not significantly above zero: in the 50ms condition, *M* = 0.001, *t*(44) = 0.15, *p* = 0.88; in the 200ms condition, *M* = -0.001, *t*(44) = -0.07, *p* = 0.94; in the 500ms condition, *M* < 0.001, *t*(44) = 0.004, *p* = 0.997; in the 1000ms condition, *M* = -0.004, *t*(42) = -0.46, *p* = 0.65. Thus, participants could hardly see the masked primes. A one-way ANOVA showed no difference of *d’*s among the four SOAs (*F* < 1.00), suggesting that SOA did not influence participants’ awareness of the masked primes (S2 File).

To probe the impact of prime visibility on the numeric judgment task, linear regressions were performed with congruency effects (i.e., AUC _incongruent_—AUC _congruent_) on *d’*s [43]. This analysis showed no correlation between the congruency effects and *d’*s in neither cued nor uncued conditions, suggesting that the observed congruency effects were not influenced by participants’ awareness of masked primes. Meanwhile, the regressions revealed significant intercepts at null *d’* in the 200ms and 500ms SOA conditions in the cued condition, and in the 200ms SOA condition in the uncued condition, suggesting that in these conditions, the subliminal semantic priming was still significant for participants who could hardly see the masked primes (Tables 5 and 6).

**Table 5:** Regressions with congruency effects (AUC) on *d*’s in the cued condition.

SOA	Predictors	Coefficients	t	P-value
**50ms**	Intercept	311.61	2.00	0.052
	*d*’	0.14	0.91	0.37
**200ms**	Intercept	1014.87	7.12	<0.001
	*d*’	-0.10	-0.67	0.50
**500ms**	Intercept	586.79	3.74	0.001
	*d*’	-0.04	-0.24	0.81
**1000ms**	Intercept	62.32	0.28	0.78
	*d*’	-0.15	-0.95	0.35

**Table 6:** Regressions with congruency effects on *d*’s in the uncued condition.

SOA	Predictors	Coefficients	t	P-value
**50ms**	Intercept	510.08	1.71	0.10
	*d*’	0.07	0.44	0.66
**200ms**	Intercept	460.35	2.11	0.04
	*d*’	0.17	1.14	0.26
**500ms**	Intercept	245.03	0.96	0.34
	*d*’	0.13	0.85	0.40
**1000ms**	Intercept	-247.38	-1.21	0.23
	*d*’	-0.03	-0.19	0.85

## Discussion

### Summary of results

This study seeks to clarify how top-down attention influences subliminal semantic processing by examining the time course of priming; as described in our predictions, top-down attention may either amplify the magnitude or modify the time course of priming, or both. Given our results, the temporal effect is salient—top-down attention delayed and prolonged the time course of subliminal semantic processing. With regard to the effect size of priming, top-down attention seems to amplify congruency effects to some extent, yet the amplification effect is relatively minor. Taken together, the results approximately support the Prediction (3)—top-down attention mostly modified the time course of subliminal semantic processing and moderately magnified priming effects.

These distinct patterns of priming between cued and uncued conditions can be explained by the cognitive resource involved. In the cued condition, participants needed additional time to process the cue, which delayed the peak of priming; however, once the cue had been processed, the facilitated top-down attention enhanced the subliminal semantic processing, leading to larger and prolonged priming effects.

Admittedly, uncued and cued conditions do not perfectly correspond to actual states of attention; disrupted attention does not guarantee complete absence of attention in the uncued condition, and facilitated attention does not mean fully-deployed attention. Meanwhile, simply contrasting congruent and incongruent trials does not tell whether the observed congruency effects resulted from facilitated responses in congruent trials or impeded responses in incongruent trials, or both; adding a control condition without primes may help in clarifying this issue. Given these limitations, whether top-down attention is as essential in subliminal semantic processing as suggested by Naccache et al. [9, 42] needs further research.

It should be noted that we employed conventional awareness test, where participants performed the same judgement task on primes as they did in the main task [43]. If participants were instead required to report the exact priming digit, such a task would have been much harder, and the response accuracy would have been even lower. This “exact report” awareness test, which is less conservative than the conventional awareness test, would underestimate participants’ awareness of primes. In the conventional awareness test, the judgment of primes may be subliminally influenced by the primes themselves. Nevertheless, if subliminally influenced judgment of primes still fails to reach an above-chance-level accuracy in the awareness test, it can be concluded that participants’ awareness of primes is nearly zero, regardless of whether there is subliminal priming or not. The subliminal priming, if any, barely helps the awareness judgment because priming mostly modulates the deviation of response trajectories (AUC) rather than changes participants’ choice. Previous research demonstrates that significant subliminal priming effects in the main task do not necessarily lead to an above-chance-level accuracy in the awareness test [11]. Many studies employing similar awareness tasks report significant priming effects at a chance-level awareness accuracy as well as non-correlation between priming effects and awareness accuracy [8, 12, 19].

### The time course of subliminal semantic processing

The impact of SOAs is substantial; congruency effects occurred earlier and decreased linearly in the uncued condition; while in the cued condition, the congruency effect was initially small but emerged robust in the middle (200ms and 500ms SOA) before eventually dissipated. Priming effects were significant when the SOA was in a moderate range (e.g., 200ms). In the 50ms and 500ms SOA conditions, the priming was significant in the cued condition while absent in the uncued condition, suggesting that the lifespan of subliminal semantic processing was extended by top-down attention. However, the current results cannot distinguish whether the extension effect is caused by the change of time course by attention or the moderate amplification effect of attention.

In addition, other evidence shows different time courses of masked priming [46]. For example, in a “same / different” judgment task, semantic priming was absent when the SOA was as short as 316ms [8]; in a spatial detection task [47], 100ms SOA and 1000ms SOA elicited opposite priming effects. Obviously, the time course of priming depends on specific tasks and stimuli, because the underlying cognitive processes are different.

### The lifespan of subliminal semantic processing

It is noteworthy that the lifespan of temporal attention window lasts longer than 1000ms, rather than a few hundreds of milliseconds suggested by Naccache et al. (2002) [9]. This is because the congruency effect was significant in the cued condition while absent in the uncued condition for the 500ms SOA; without assistance from top-down attention, subliminal semantic processing disappeared when the SOA was as long as 500ms. Thus, to sustain subliminal semantic processing, the temporal attention window should remain open for at least 500ms after the onset of primes. Since the onset of the cue was 484ms prior to the onset of primes (200ms for the cue and 284ms for the pre-mask), the temporal attention window remained open for at least 1000ms to sustain priming (200ms cue + 284ms pre-mask + 500ms SOA + some response latency).

### The relationship between cognitive control and unconscious processing

The conventional view is that conscious and unconscious processing is distinct, and being conscious of something means attending to it, vice versa. Conscious processing is assisted by attention, whereas subliminal processing is diffuse, parallel, and free from cognitive control [48, 49]. The involvement of consciousness can even impede subliminal processing because attention selectively suppresses autonomous processing. Thus, consciousness tends to restrict rather than assist subliminal processing [1–4].

However, later studies highlights the distinction between consciousness and attention—consciousness is subjective experience while attention is the mechanism allocating limited cognitive resource [6]. Recent evidence demonstrates that attention influences subliminal processing [7, 9, 42, 50, 51]. The present study adds further evidence to the robustness of elaborate subliminal processing with little assistance from attention. In brief, attention influences both conscious and unconscious processing, and is necessary for consciousness while unnecessary for unconscious processing.

More importantly, the elaborate and flexible nature of subliminal processing revealed by recent research makes the boundary between conscious and unconscious processing vague [5, 50, 51]. Top-down attention does influence the time course of subliminal semantic processing, and the mechanism underlying the interplay of top-down attention and subliminal processing has been explained by the global workspace theory [52]. This theory differentiates two major computational spaces in the brain: peripheral processing space and global workspace; the peripheral processing space consists of autonomous and functionally specialized processors, which project their processing to the global workspace; the attentional system in the global workspace selectively polarizes or suppresses some autonomous processors by allocation limited cognitive resource [52]. The gating mechanism of top-down attention selectively amplifies or attenuates signals from peripheral processors in the global workspace. The autonomous activities of peripheral processors explain spontaneous elaborate processing without awareness: some modular processors are specialized in elaborate processing such as semantic categorization, numerical representation, facial recognition, and scene comprehension. These processors automatically process inputs and send outputs to the global workspace; some outputs are not salient enough to be attended in the global workspace. Attended processing gains more cognitive resource in the global workspace and result in extended subliminal priming, while subliminal processing without assistance of attention quickly fades away in the global workspace [5, 42, 48, 52]. Given the link between cognitive control and subliminal processing, the dichotomous view that consciousness and unconsciousness are distinct seems outdated. States of consciousness are more likely on a continuum, with full consciousness at one end and unconsciousness (e.g., sleep, coma) at the other end [53, 54].

## Conclusions

In conclusion, top-down attention modifies the time course of subliminal semantic processing and the temporal attention window lasts more than 1000ms. The amplification effect of attention is relatively minor.

## Supporting information

S1 File Supplementary plots. (DOCX)

S2 File Full data. (XLSX)
