# Supplementary material for: The role of attention in subliminal semantic processing: A mouse tracking study
Source: PLoS One. 2017 Jun 13;12(6):e0178740. doi: 10.1371/journal.pone.0178740 (PMC5469464; doi:10.1371/journal.pone.0178740)
Supplement: S1 File — (DOCX) [file pone.0178740.s001.docx]

# Supplementary plots

The figures show average raw trajectories of x-coordinates over raw response time. Specifically, the y-axis represents the x-coordinates of the cursor on the screen while the x-axis represents the raw time of responses. The x-coordinates are the horizontal distance between the cursor position and the initial position (horizontal center on the screen) and is measured by the number of pixels. The response timestamps on the y-axis range from 0 to 1200ms (the average response time is 893ms and the standard deviation is 192ms). The average raw trajectories are plotted separately in cued and uncued conditions for each SOA condition (50ms, 200ms, 500ms, 1000ms); the red trajectories represent congruent trials while the green trajectories represent incongruent trials. The shaded ribbons (gray area) represent confidence intervals circumscribed by two standard errors (one standard error on each side).

# 50ms SOA: X position

| 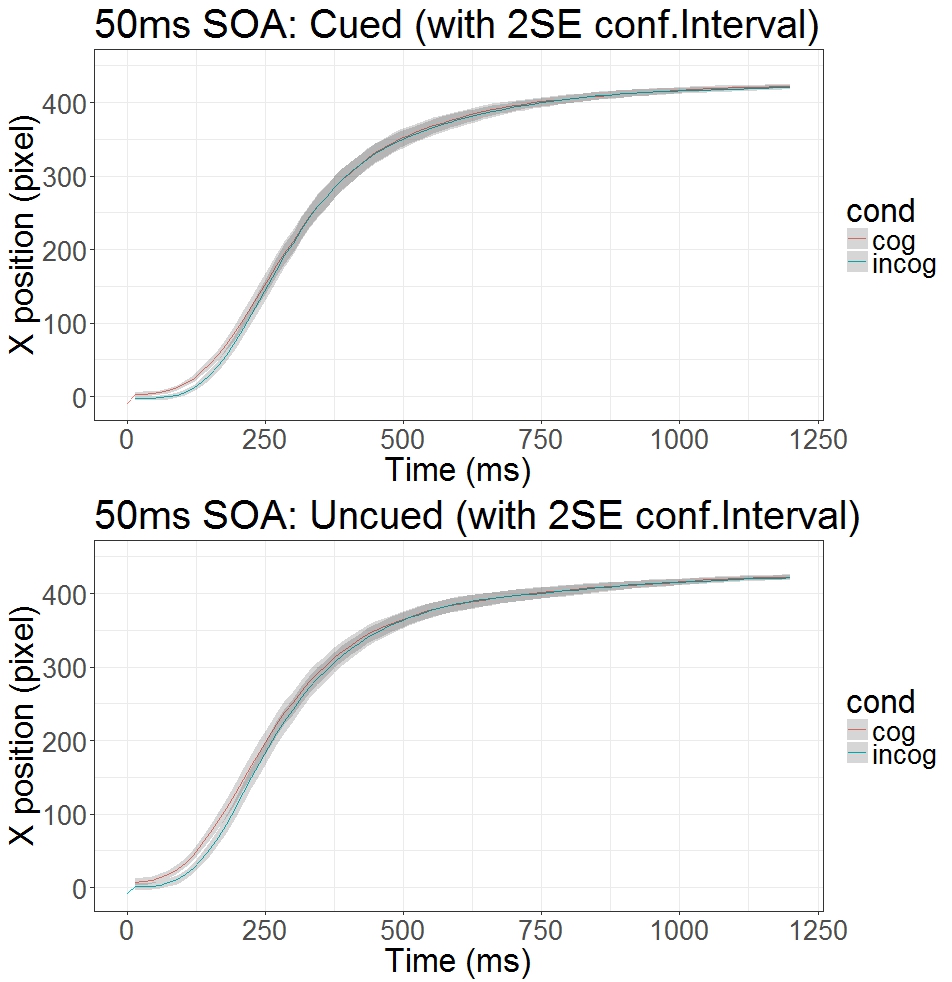 |
| --- |

# 200ms SOA: X position

| 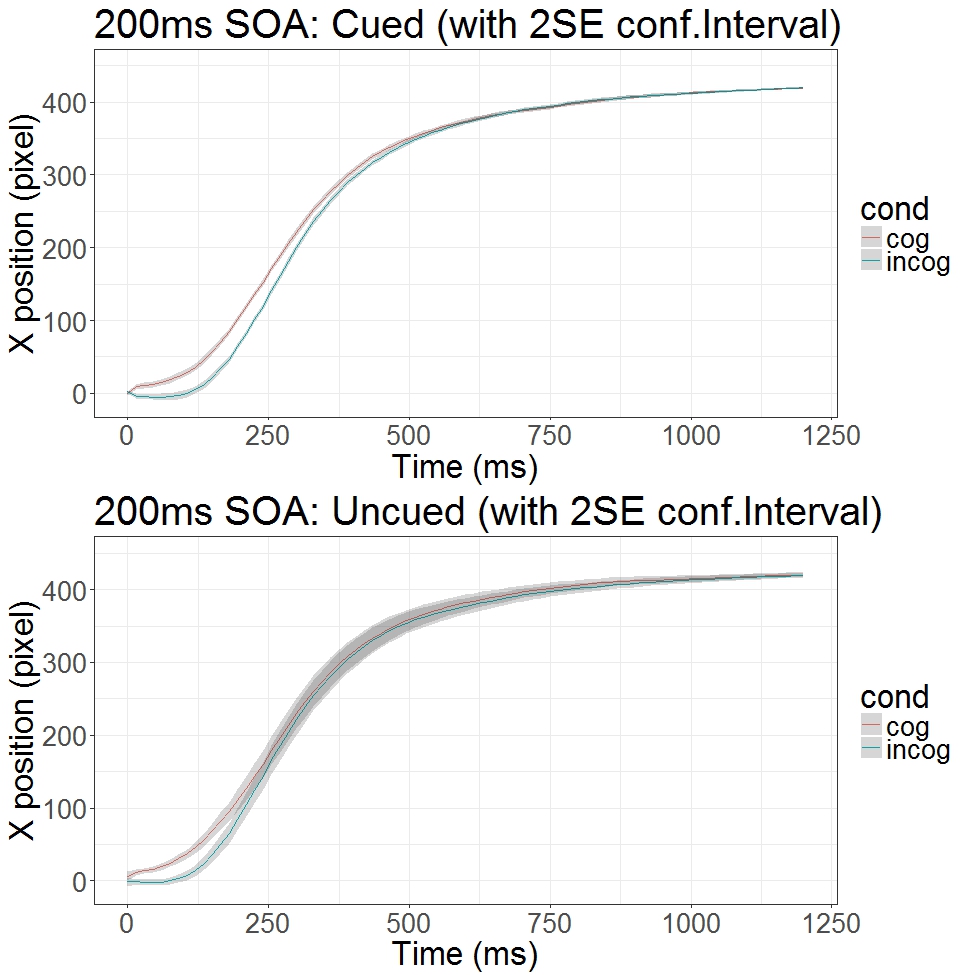 |
| --- |

| 500ms SOA: X position  \| 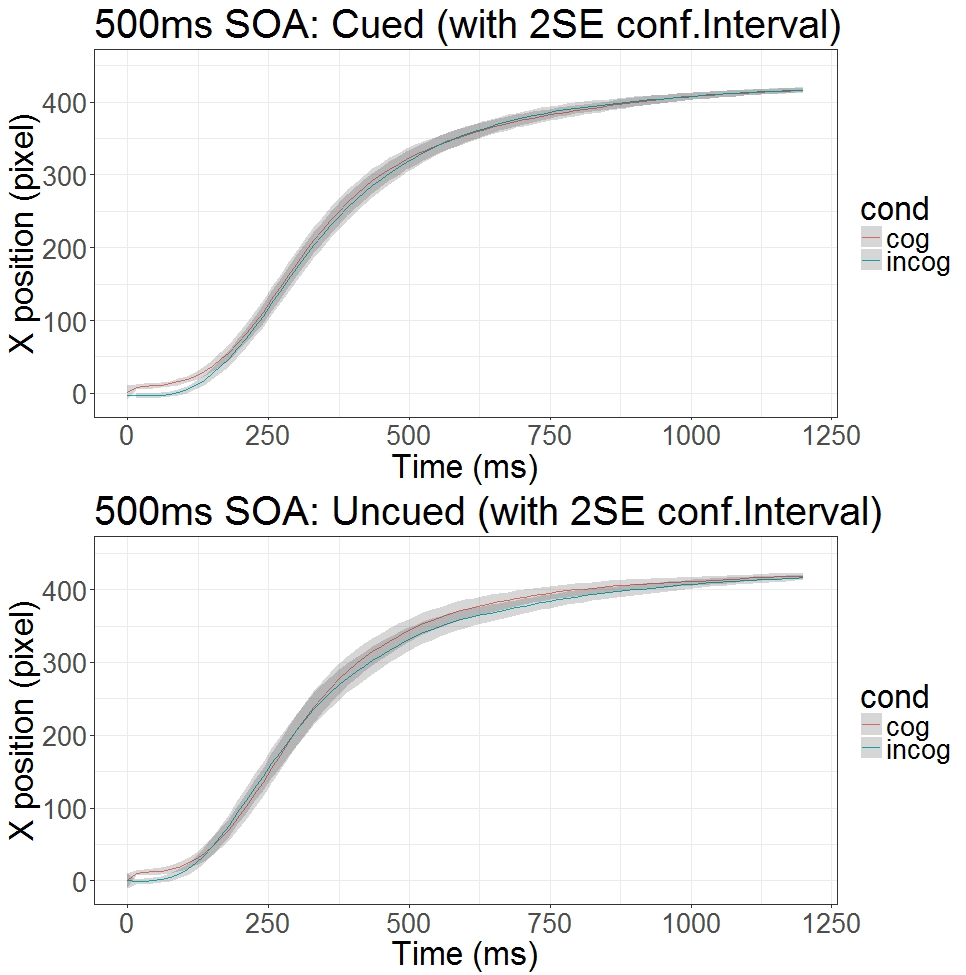 \| \| --- \| |
| --- | --- |
|  |
| 1000ms SOA: X position  \| 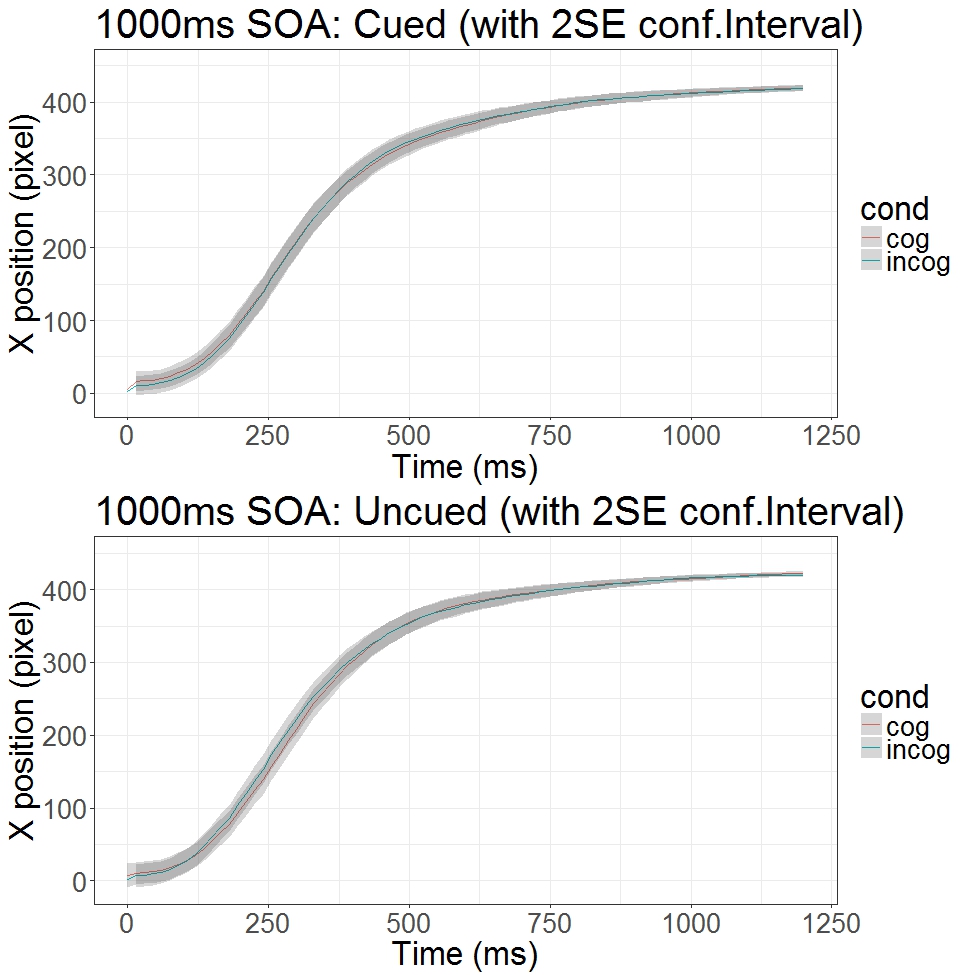 \| \| --- \| |
